# Closer to 90–90–90. The cascade of care after 10 years of ART scale-up in rural Malawi: a population study

**DOI:** 10.7448/IAS.19.1.20673

**Published:** 2016-02-15

**Authors:** David Maman, Benson Chilima, Charles Masiku, Ahidjo Ayouba, Sophie Masson, Elisabeth Szumilin, Martine Peeters, Nathan Ford, Annette Heinzelmann, Benjamin Riche, Jean-François Etard

**Affiliations:** 1 Epicentre, Paris, France; 2 Ministry of Health, Lilongwe, Malawi; 3 Médecins Sans Frontières, Lilongwe, Malawi; 4 UMI 233 TransVIHMI, Institut de Recherche pour le Développement (IRD), Montpellier 1 University, Montpellier, France; 5 Médecins Sans Frontières, Paris, France; 6 Centre for Infectious Disease Epidemiology and Research, University of Cape Town, Cape Town, South Africa; 7 Service de Biostatistique, Hospices Civils de Lyon, Lyon, France; 8 Université de Lyon, UMR CNRS 5558, Lyon, France; 9 Laboratoire de Biométrie et Biologie Evolutive, Equipe Biotatistique-Santé, CNRS UMR5558, Villeurbanne, France

**Keywords:** HIV, incidence, prevalence, viral load

## Abstract

**Introduction:**

The antiretroviral therapy (ART) programme supported by Médecins Sans Frontières in the rural Malawian district of Chiradzulu was one of the first in sub-Saharan Africa to scale up ART delivery in 2002. After more than a decade of continuous involvement, we conducted a population survey to evaluate the cascade of care, including population viral load, in the district.

**Methods:**

A cross-sectional household-based survey was conducted between February and May 2013. Using a multistage cluster sampling method, we recruited all individuals aged 15 to 59 years living in 4125 randomly selected households. Each consenting individual was interviewed and tested for HIV at home. All participants who tested positive had their CD4 count and viral load measured. The LAg-Avidity assay was used to distinguish recent from long-term infections. Viral suppression was defined as a viral load below 1000 copies/mL.

**Results:**

Of 8271 individuals eligible for the study, 7269 agreed to participate and were tested for HIV (94.1% inclusion for women and 80.3% for men). Overall HIV prevalence and incidence were 17.0% (95% CI 16.1 to 17.9) and 0.39 new cases per 100 person-years (95% CI 0.0 to 0.77), respectively. Coverage at the other steps along the HIV care cascade was as follows: 76.7% (95% CI 74.4 to 79.1) had been previously diagnosed, 71.2% (95% CI 68.6 to 73.6) were under care and 65.8% (95% CI 62.8 to 68.2) were receiving ART. Finally, the proportion of participants who were HIV positive with a viral load ≤1000 copies/mL reached 61.8% (95% CI 59.0 to 64.5).

**Conclusions:**

This study demonstrates that a high level of population viral suppression and low incidence can be achieved in high HIV prevalence and resource-limited settings.

## Introduction

UNAIDS recently set up an ambitious 90–90–90 targets, with the objective that by 2020 90% of all people living with HIV will know their status, 90% of those diagnosed will receive sustained antiretroviral therapy (ART) and 90% of those on ART will reach undetectable viral load [1]. Despite wider ART availability in recent years, uncertainty remains whether such levels of population viral suppression can be achieved, especially in resource-limited settings. UNAIDS has estimated that only 27% of the people living with HIV in sub-Saharan Africa were receiving ART in 2012 [2]. In the United States, among all persons living with HIV in 2009, only 25% had a suppressed viral load [3].

The cascade of care is a conceptual representation of the effectiveness of a cascade of services from testing, linkage and retention in care and to ART initiation and sustained virological suppression [4,5]. Measuring each step of this continuum of care is increasingly seen as a critical way to assess programme effectiveness. It also provides an opportunity to evaluate what the population impact of universal ART treatment would be on the cascade and on ART eligibility, as recommended by the World Health Organization (WHO) since September 2015 [6].

To help understand what can be achieved in high prevalence settings in sub-Saharan Africa, an HIV programme supported by Médecins Sans Frontières (MSF) in a rural district of Malawi is of particular interest because of its duration and history. It is located in the district of Chiradzulu, in southern Malawi, where HIV prevalence was estimated at 14.5% in 2010 [7]. This was the first programme to provide ART in public facilities in the country. It has been scaled up since 2002 to facilitate access to treatment [8]. To achieve this objective, the programme was decentralized to peripheral facilities to provide easier access to care. To cope with the scarcity of skilled human resources, it implemented task-shifting, permitting nurses to initiate ART, which was generalized to the country [9]. In 2012, 27,000 patients (out of a population of 270,000 people) were receiving ART in the district.

In order to assess programme effectiveness after 10 years of ART scale-up, we performed a population-based survey to measure HIV incidence and coverage of each step of the cascade of care.

## Methods

### Study design and ethics

The 2012 Chiradzulu HIV Impact in Population Survey is a district representative household-based survey that was conducted between February and May 2013. The sampling frame used was the 2009 Malawi Population and Housing Census, provided by the Statistical Office. A two-stage design was used to randomly select 4125 households. One cluster was a census enumeration area. Among the 335 census enumeration areas in the Chiradzulu district, 165 were selected during the first stage, with a probability to be selected proportional to the number of households in each enumeration area. Then, 25 households were randomly selected in each cluster. Each resident of the selected households aged 15 to 59 was considered eligible for the study.

Ethical approval was obtained in Malawi from the National Health Sciences Research Committee (ref. 1085) and in France from the Comité de Protection des Personnes d'Ile de France (ref. 12084). Each participant was informed of the study objectives and procedures and provided written consent to participate in the study and be tested for HIV prior to starting the interview.

### Procedure

Community mobilization for the survey was performed as a two-step process. With the help of the MSF Information, Education and Community team who had been working in the district for 15 years, the study team met with local leaders a few weeks prior to the start of the study. Furthermore, each cluster selected was visited by the study team up to two weeks prior to the study to directly mobilize the community. Leaflets were distributed to inform the community about the survey. Patients were told that the survey was about HIV and that they would be tested for HIV if they agreed to participate.

The questionnaire was based on the MACRO questionnaire framework used for the Demographic and Health Survey to ensure maximum transportability of the results [10,11]. The questionnaire was translated into Chichewa, the main language spoken in the district, and back-translated. The questionnaire was pretested in January 2013. The individual questionnaires collected socio-economic and behavioural information. Men self-reported their circumcision status using graphic tools and women reported their previous births, antenatal care and, if HIV positive, their PMTCT care. Participants were asked to report their history of HIV testing and the result of their last test. If HIV positive, participants were asked about awareness of their HIV status and about ART use.

A serial rapid-testing algorithm was used to test participants for HIV. Determine Rapid HIV-1/2 Antibody (Abbott Laboratories, Abbott Park, IL, USA) was followed by a Unigold Rapid HIV Test (Trinity Biotech PLC, Bray, Ireland). In case of discordant results, an ELISA test was used to confirm infection (Genetic Systems HIV-1/HIV-2 Plus O EIA, Bio-Rad, Redmond, WA, USA). From all HIV-positive participants, a venous blood sample was collected for home-based CD4 count (PIMA CD4 counter, Alere PIMA, Jena, Germany). Viral load (G2 real-time PCR, Biocentric^®^, Bandol, France) and assay for recent infection using the limiting antigen avidity EIA test (LAg test, Sedia Biosciences, Portland, OR, USA) were performed at the Institut de Recherche pour le Développement (IRD) in Montpellier, France. The LAg test is a serological assay that detects increasing avidity antibody maturation following seroconversion. It is used for distinguishing long-term HIV-1 infections from recent ones. It identifies infections for which seroconversion occurred during the past 130 days (95% CI 118 to 142). To improve accuracy and reduce misclassification we used a multiple assay algorithm. To be classified as recently infected, a participant must (1) have a normalized optical density threshold of 1.5 or below, (2) not report being on ART and (3) have a detectable viral load. A false recent rate (FRR) of 0.5% (95% CI 0.2 to 0.8) was used to account for misclassification. We defined the different steps of the cascade using WHO-recommended metrics [12]. Diagnostic, linkage and retention in care and ART use were all self-reported by participants. HIV awareness was defined as a history of first positive HIV test prior to the survey. Linkage to care was defined as one medical contact for HIV care after a positive test. Retention in care was defined as an HIV-related medical consultation within the last six months. ART use was self-reported by the HIV-positive participants, who were also asked to show their treatment to the nurse. ART eligibility was defined following the 2011 Malawian ART guidelines [13]. An HIV-positive individual was considered eligible for ART if he or she had ever started ART, if he or she had a CD4 count below 350 cells/µL or (for women) if she reported being pregnant or breastfeeding. Viral suppression was defined as a viral load below 1000 copies/mL.

### Data management and analysis

Using EpiData Entry 3.1 software (EpiData Association, Odense, Denmark), all data were anonymized before being double-entered and checked. They were analyzed using Stata 13 (StataCorp, College Station, TX, USA). Descriptive statistics were weighted to account for the selection probability of the cluster sampling procedure and are presented with their 95% confidence intervals. We estimated HIV incidence as the number of new infections per 100 person-years using the estimator described by Kassanjee, McWalter and Welte (www.incidence-estimation.org/page/tools-for-incidence-from-biomarkers-for-recent-infection) [14]. Because the FRR of the recent infection testing algorithm was not known for the Chiradzulu population in 2013, we conducted a sensitivity analysis applying the FRR at 0 and 1%. Two-sample t-test and Pearson chi-square statistics were used to compare continuous and categorical descriptive outcomes, respectively. The prevalence-to-incidence ratio reports the number of prevalent cases per incident case [15].

## Results

**Figure 1:**
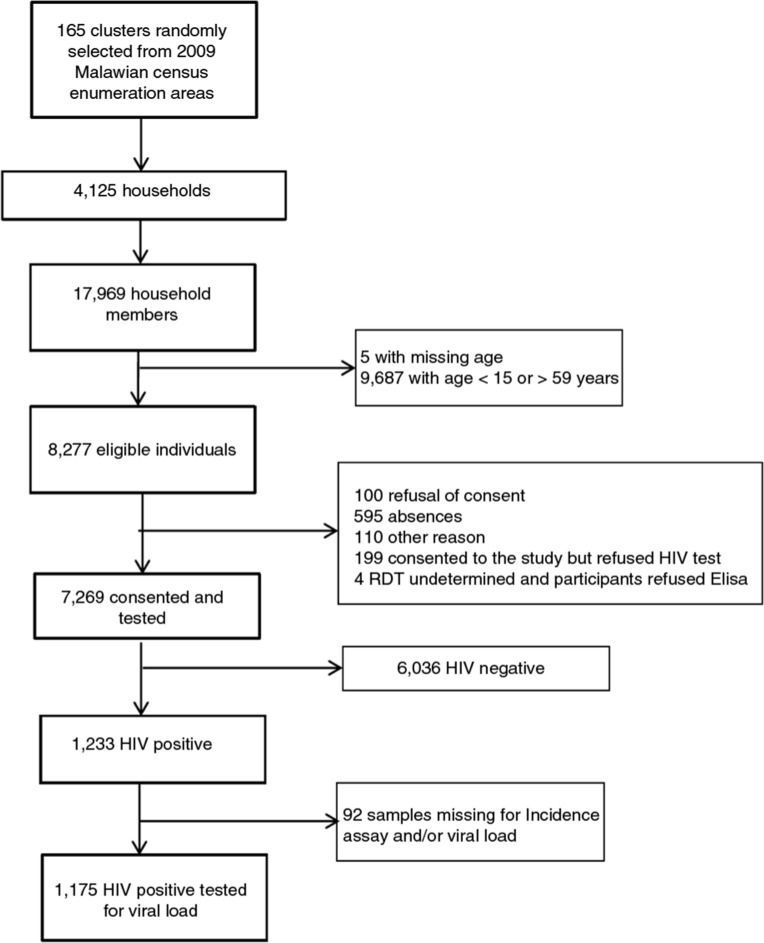
Study flow chart.

The study was conducted between February and May 2013 and collected information on 4125 households. Of the 8277 individuals 15 to 59 years of age eligible for the study, 7269 (87.8%) signed the written consent, completed the individual questionnaire and provided blood for the HIV test (Figure 1). Inclusion was higher for women than men (94.0% vs. 80.3%). Median age was 29 years [IQR 21 to 39] for women and 28 years [IQR 19 to 38] for men. Of the participants, 95.4% (95% CI 94.9 to 95.9) had been residents of Chiradzulu District for more than 10 years.

**Figure 2:**
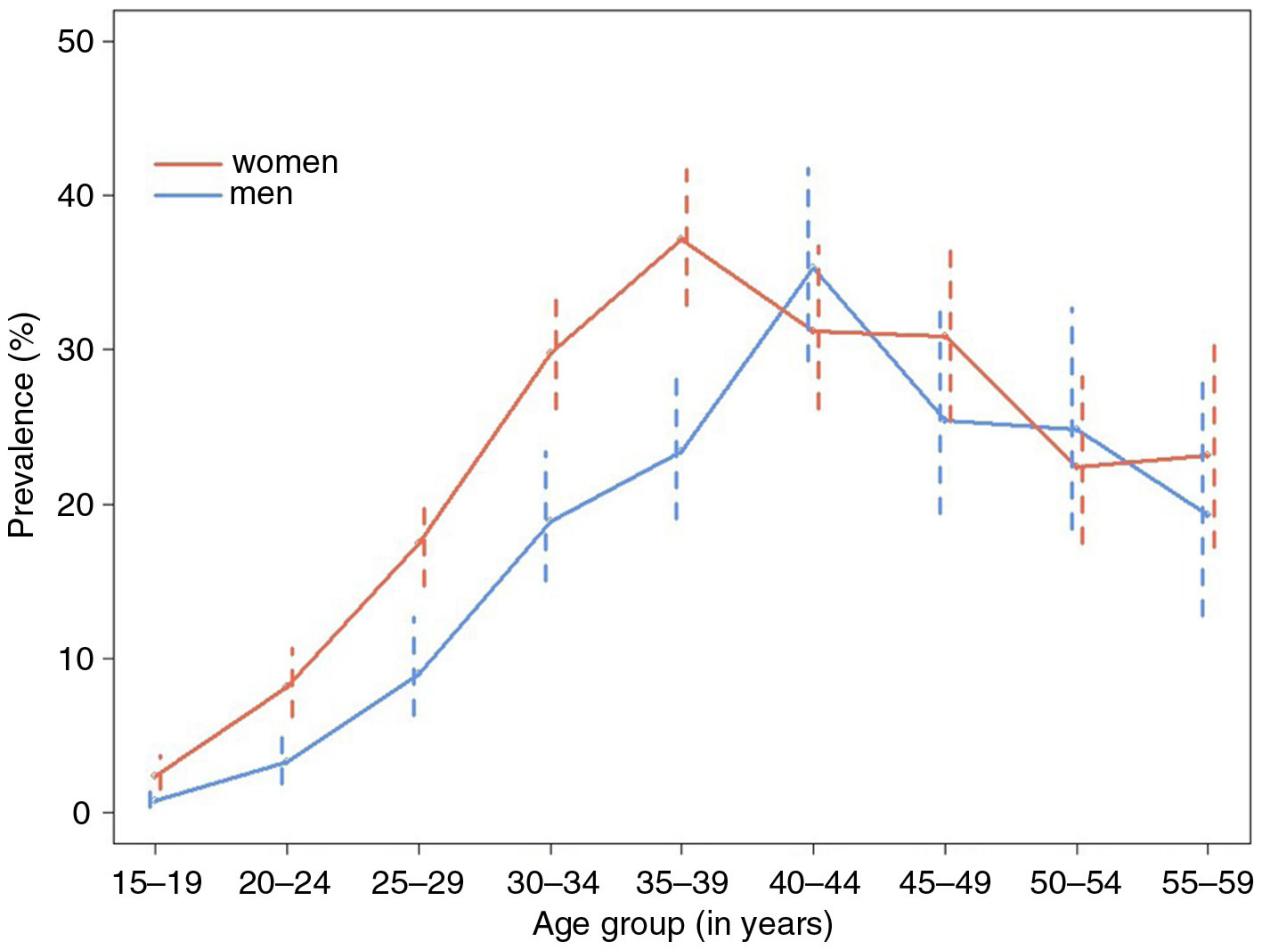
Weighted HIV prevalence among men and women in Chiradzulu, Malawi, 2013.

Of the 7269 participants tested for HIV, 1233 were found to be HIV positive (Table 1). The overall weighted prevalence was 17.0% (95% CI 16.1 to 17.9). Prevalence was higher for women than men (19.7 vs. 13.0, *p* < 0.01). HIV prevalence increased with age, with a maximum at 35 to 39 for women and 40 to 44 for men (Figure 2). For women, prevalence was significantly higher at age 35 to 39 years than 30 to 34 (37.2% vs. 29.8%, *p*=0.01) and for those not pregnant or breastfeeding compared to those who were (22.9% vs. 13.6%, *p*<0.01). Prevalence was higher among widows (54.4%, 95% CI 48.1 to 60.6) than among married participants (18.5%, 95% CI 17.3 to 19.7) or among those who never married (2.7%, 95% CI 2.0 to 3.6).

**Table 1:** Characteristics of participants and prevalence

	Total tested	Number of HIV-positive individuals	Weighted HIV prevalence (% + 95% CI)
Sex			
Female	4274	839	19.7 (18.5 to 21.0)
Male	2995	394	13.0 (11.8 to 14.4)
Age (years)			
15 to 19	1563	28	1.6 (1.1 to 2.3)
20 to 24	1120	67	6.2 (4.9 to 8.0)
25 to 29	1066	154	14.4 (12.3 to 16.7)
30 to 34	979	249	25.7 (22.9 to 28.6)
35 to 39	828	266	31.8 (28.6 to 35.2)
40 to 44	564	186	32.9 (29.0 to 37.1)
45 to 49	441	127	28.8 (24.6 to 33.5)
50 to 54	411	96	23.3 (19.3 to 27.9)
55 to 59	297	60	21.7 (17.1 to 27.1)
Marital status (missing: 30)			
Never married	1703	48	2.7 (2.0 to 3.6)
Married/living together	4648	859	18.5 (17.3 to 19.7)
Divorced/separated	626	179	29.2 (25.6 to 33.1)
Widowed	262	141	54.4 (48.1 to 60.6)
Education (missing: 5)			
Primary or less	5444	1047	19.4 (18.4 to 20.6)
Secondary or less	1820	185	9.8 (8.5 to 11.3)
History of HIV testing (missing: 3)			
Never tested	1740	98	5.7 (4.7 to 7.0)
Ever tested	5526	1135	20.5 (19.4 to 21.7)
Residence in Chiradzulu (missing: 4)			
< 10 years	320	67	21.2 (16.8 to 26.3)
≥ 10 years	6945	1165	16.8 (15.9 to 17.7)
Mobility (nights outside/month) (missing: 4)			
0	5461	911	16.7 (15.6 to 17.7)
1 to 5	1602	276	17.4 (15.5 to 19.4)
6 +	202	46	23.2 (17.6 to 29.9)
Pregnant or breastfeeding^a^			
Yes	1444	194	13.6 (11.8 to 15.6)
No	2830	1250	22.9 (21.3 to 24.6)
Medical circumcision^b^ (missing: 15)			
Yes	295	2	0.75 (0.2 to 3.2)
No	2651	293	14.4 (13.6 to 15.9)
TOTAL	7269	1233	17.0 (16.1 to 17.9)

a Only women;

b only men.

A total of 13 HIV-positive individuals (12 women and 1 man) were classified as recently infected, corresponding to an incidence of 0.35 new cases per 100 person-years (95% CI 0.0 to 0.72) and a prevalence-to-incidence ratio of 44:1. We performed sensitivity analysis varying the FRR of the LAg test. A FRR of 0% would correspond to an incidence of 0.63 new cases per 100 person-years, whereas a FRR of 1% would give an incidence of 0.06 new cases per 100 person-years.

**Figure 3:**
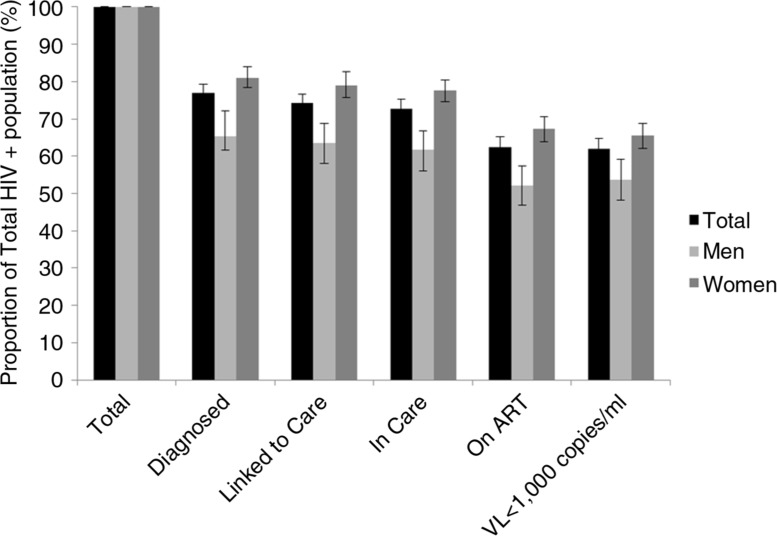
HIV cascade of care, Chiradzulu, Malawi, 2013.

Of the 1233 participants found to be HIV positive, a total of 1174 (95.1%) had completed the individual questionnaire and had their CD4 cell count and HIV load ascertained. Among them, 77.0% (95% CI 74.4 to 79.3) reported being already diagnosed for HIV (Figure 3). The proportion of the HIV-positive individuals ever linked to care was almost equivalent at 74.2% (95% CI 72.5 to 76.7). Retention in care was also high at 72.8% (95% CI 70.1 to 75.3), indicating the losses after linkage to care were small. The loss between those under care and on ART was greater, with a proportion of HIV-positive individuals on ART at 62.5% (95% CI 59.7 to 65.3). Finally, population viral suppression was at 61.9% (95% CI 58.9 to 64.7). Sensitivity analysis revealed mild differences in population viral suppression dependent on the threshold used for defining viral suppression. Figures would be lower, at 57.7% (95% CI 54.7 to 60.6) and 55.5% (95% CI 52.5 to 58.4) with a threshold at 300 and 200 copies/mL, respectively, and higher at 65.8% (95% CI 62.9 to 68.6) with a threshold at 5000 copies/mL. The cascade of care showed important gender differences, starting from diagnosis where women were more likely than men to be diagnosed for HIV (81.3% vs. 67.1%, *p*<0.01). This difference remained stable throughout the cascade of care, with viral suppression rates of 65.5% and 53.7% for women and men (*p*<0.01), respectively.

**Figure 4:**
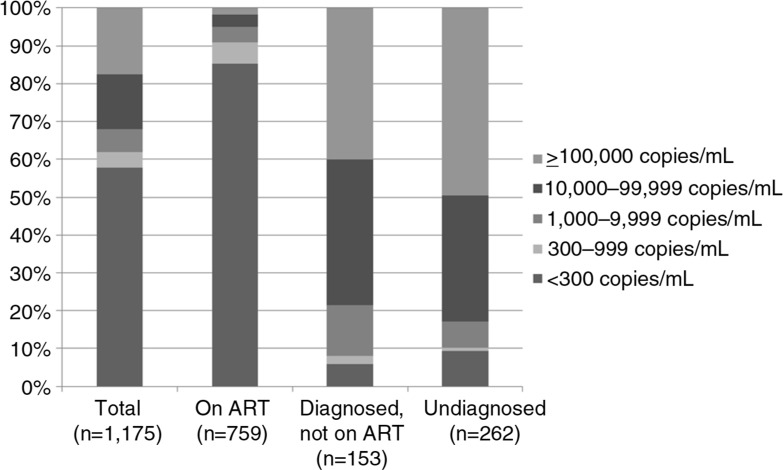
Population viral load distribution by ART and diagnostic status.

The distribution of viral load among HIV-positive participants on ART, diagnosed but not on ART and undiagnosed is presented in Figure 4. Among those on ART (median time on ART 3.9 years [1.5 to 5.7]), the proportion of participants with VL <1000 copies/mL was 90.8% (95% CI 88.5 to 92.7) and only 1.7% (95% CI 1.0 to 3.1) had a viral load ≥100,000 copies/mL. As expected, viral load among those not receiving treatment was much higher. Of those previously undiagnosed, 49.6% (95% CI 43.3 to 56.0) had a viral load ≥100,000 copies/mL, a figure higher than among those who were diagnosed but not on ART (40.1%, 95% CI 32.5 to 48.3), although the difference in distribution between those two categories was only marginally significant (*p*=0.08).

Using the previous 2011 Malawian guidelines, we estimated that 80.0% (95% CI 77.6 to 82.3) of the HIV-positive population was in need of ART at the time of the survey (Table 2). The WHO 2013 ART guidelines, which were implemented in 2014 in Malawi (raising the CD4 threshold from 350 to 500 CD4 cells/µL), increased eligibility to 87.5% (95% CI 85.5 to 89.4) and decreased ART coverage from 80.5% (77.8 to 83.0) to 73.6% (95% CI 70.7 to 76.1). The increase in eligibility would be higher in men (75.9% to 87.1%) than in women (81.9% to 87.8%).

**Table 2:** ART eligibility and coverage by guideline (% + 95% CI)

	Malawi guidelines during the survey (CD4 ≤350 cells/µL)	WHO 2013 guidelines (CD4 ≤500 cells/µL)	WHO 2015 guidelines (universal ART)
ART eligibility			
Women	81.9 (78.9 to 84.5)	87.8 (85.2 to 90.0)	100
Men	75.9 (71.0 to 80.2)	87.1 (83.1 to 90.3)	100
Total	80.0 (77.6 to 82.3)	87.5 (85.5 to 89.4)	100
ART coverage			
Women	83.9 (80.9 to 83.6)	78.3 (75.0 to 81.2)	67.3 (95% CI 63.9–70.5)
Men	72.1 (66.2 to 77.4)	62.9 (57.1 to 68.3)	52.1 (95% CI 46.9–57.3)
Total	80.5 (77.8 to 83.0)	73.6 (70.7 to 76.1)	64.4 (61.5 to 67.2)

## Discussion

In this population study, we found that almost two-thirds of the HIV-positive individuals living in the district of Chiradzulu achieved a viral load <1000 copies/mL, 10 years after scaling up ART services and a median time on ART of almost four years.

The study demonstrates that a high proportion of population viral suppression, which is associated with a substantial decrease in HIV incidence, can be achieved in resource-limited settings. Of the total HIV positive population, 77% were diagnosed, 62.5% were on ART and 61.9% were virally suppressed. Our data suggest that the targets recently set by UNAIDS, which aim at 90% of those who are HIV positive to be diagnosed, 81% under ARV and 73% suppressed are close to being achieved in Chiradzulu district. The proportion of HIV positive individuals who are virologically suppressed is higher than other reports from sub-Saharan Africa, 50% higher than figures recently reported from population studies in Swaziland or in South Africa, which estimated viral suppression among all HIV-positive individuals (regardless of ART use) at 35% and 40%, respectively [16,17]. It is also higher than levels of viral suppression found in developed countries. In one study from British Columbia, Canada, the proportion of HIV-positive individuals with viral suppression reached 34.6% in 2011 [18]. However, in this study, population viral suppression was estimated from routine data and the threshold for viral suppression was much lower (<50 copies/mL). In our study, we used a representative sample of the general population. It allowed us to estimate the viral load of all HIV-positive individuals, including those not under care or undiagnosed, to provide a more accurate estimate of the viral suppression at the population level [19]. Furthermore, sensitivity analysis showed that using a lower threshold of 200 copies/µL to define viral suppression only marginally changed the estimate.

This outcome was achieved in a simplified programme where routine viral load monitoring was not implemented at the time of the survey and where no universal testing campaign had ever been organized. This programme was one of the first in sub-Saharan Africa to be scaled up 10 years ago, and with the long lasting support of MSF and the national ART programme (from 2004 onwards), continuous decentralization of care, spacing of medical visits and task-shifting have allowed more and more individuals to access ARV freely, close to where they live without interruption of the drug supply [8,9,20–22]. Two main reasons can be put forward to explain the high level of population viral suppression in Chiradzulu: the performance of individuals receiving ART and the high number of individuals eligible for ART. First, 90% of HIV-positive individuals receiving ART had a viral load lower than 1000 copies/mL, similar to the proportion found in a previous study performed in the district in 2004 [8]. This result illustrates the capacity of the programme to maintain high adherence among patients receiving ART. Another likely explanation for the high proportion of HIV-positive individuals suppressed in the population is the surprisingly high proportion of HIV-positive individuals in need of ART in Chiradzulu. Indeed, we found that 80% of the HIV-positive participants were eligible for ART (either on ART or in need of ART) according to the 2011 national ART guidelines [13], almost twofold higher than the national estimate of 44% in 2013, using the Spectrum model [2]. However, ART eligibility, at the national level and in Chiradzulu district, has never been directly measured in Malawi but only estimated using models. Our study found that implementing the 2013 WHO guidelines would only lead to an 8% eligibility increase in Chiradzulu, an estimate much lower than those produced by models. The high proportion of individuals eligible for ART could be partially explained by the implementation, in 2011, of the PMTCT B+ option in the country, which has led to a massive increase in ART initiation as, with this option, HIV-positive pregnant or breastfeeding women become eligible for ART regardless of their CD4 status [23].

Another potential explanation for this high proportion of eligible individuals is linked to a key finding of the study: HIV incidence. Compared to other studies that directly measured HIV incidence in the population, the prevalence-to-incidence ratio in Chiradzulu was more than four times higher (44:1) than those estimated during the national population surveys implemented in Kenya and Swaziland, which found rates of 9:1 and 8:1, respectively [24–26]. Decreasing incidence in Chiradzulu could partially explain why ART eligibility and the proportion of people diagnosed were high in the district. Indeed, as new cases are less likely to be eligible for ART due to high CD4, we can expect the proportion of HIV-positive individuals who are ART eligible to increase when incidence is decreasing [27].

These data show that implementing the new 2015 WHO guidelines, recommending universal access to ART regardless of CD4 count, would not be as costly as expected because at the time of the survey, in 2013, under the 350 CD4 cell count threshold for treatment initiation, 80% of the HIV-positive population was already eligible.

This study also provides an estimation of the cascade of care in the district. We found more than three-quarters of HIV-positive individuals were previously diagnosed, a proportion similar to estimates from developed regions like British Colombia in Canada and much higher than national surveys from Kenya, Swaziland and South Africa [18,25,26,28]. These findings suggest that a high proportion of the HIV-positive population can be identified in high HIV prevalence and limited-resource settings in sub-Saharan Africa. However, we found that men were less likely than women to be diagnosed for HIV. This observation confirms earlier findings from other studies in the region and highlights the need to improve access to HIV testing for men [25,28]. The second most important loss in the continuum of care is the proportion of HIV-positive individuals under care who are not receiving ART. Indeed 72% of the HIV-positive population was under care at the time of the survey but only 62% reported being on ART. These data suggest that the application of the 2015 WHO recommendations advising universal ART regardless of CD4 threshold would have an immediate and significant impact on the continuum of care.

Of note, we found that HIV prevalence among the widowed population was high, above 50%. This finding suggests that a high proportion of their partners died from HIV, which is the leading cause of mortality among adults in Malawi.

Our study has limitations. Non-response to the survey, especially in men, may have biased our estimates [29–31]. Another limitation of our study is its cross-sectional design, which prevented us from drawing a causal relation between HIV incidence and high population viral suppression. Nevertheless, we suspect the low incidence is at least partially the result of the high ART coverage. The contribution of ART to reducing HIV incidence at the population level is currently being assessed by several ongoing clinical trials [32,33]. Furthermore, this study was the first of this type performed in the district and as such, we cannot provide information on the trend of HIV incidence. All data except laboratory tests were self-reported. Another limitation to note is that we did not include individuals aged 60 and above, and the very high (>20%) prevalence among those aged 55 to 59 years suggests a significant number of HIV-positive individuals in these higher age groups. Finally, as incidence was lower than we expected, we did not have sufficient incident cases to stratify it by other covariates.

In conclusion, our study provides new evidence on the feasibility of achieving a high proportion of viral suppression at the population level and low HIV incidence in high prevalence settings in sub-Saharan Africa. Universal ART regardless of CD4 count and increasing diagnosis among men would have significant impact in reaching the 90–90–90 targets.
